# Cytotoxicity Analysis and In Silico Studies of Three Plant Extracts with Potential Application in Treatment of Endothelial Dysfunction

**DOI:** 10.3390/pharmaceutics15082125

**Published:** 2023-08-11

**Authors:** Andreea Roxana Ungureanu, Violeta Popovici, Camelia Oprean, Corina Danciu, Verginica Schröder, Octavian Tudorel Olaru, Dragoș Paul Mihai, Liliana Popescu, Emanuela-Alice Luță, Carmen Lidia Chițescu, Cerasela Elena Gîrd

**Affiliations:** 1 Faculty of Pharmacy, “Carol Davila” University of Medicine and Pharmacy, 6 Traian Vuia Street, 020956 Bucharest, Romania; andreea.ungureanu@drd.umfcd.ro (A.R.U.); octavian.olaru@umfcd.ro (O.T.O.); liliana.costea@drd.umfcd.ro (L.P.); emanuela.luta@drd.umfcd.ro (E.-A.L.); cerasela.gird@umfcd.ro (C.E.G.); 2 Department of Microbiology and Immunology, Faculty of Dental Medicine, Ovidius University of Constanta, 7 Ilarie Voronca Street, 900684 Constanta, Romania; popovicivioleta@gmail.com; 3 Faculty of Pharmacy, “Victor Babeş” University of Medicine and Pharmacy, 2 Eftimie Murgu Street, 300041 Timisoara, Romania; corina.danciu@umft.ro; 4 OncoGen Centre, County Hospital’ Pius Branzeu’, Blvd. Liviu Rebreanu 156, 300723 Timisoara, Romania; 5 Department of Cellular and Molecular Biology, Faculty of Pharmacy, Ovidius University of Constanta, 6 Capitan Al. Serbanescu Street, 900001 Constanta, Romania; verginica.schroder@univ-ovidius.ro; 6 Faculty of Medicine and Pharmacy, “Dunărea de Jos” University of Galați, A.I. Cuza 35, 800010 Galați, Romania; carmen.chitescu@ugal.ro

**Keywords:** cytotoxicity, endothelial cells, brine shrimp lethality assay, *Calendulae flos* extract, *Ginkgo bilobae folium* extract, *Sophorae flos* extract, molecular docking, caspases

## Abstract

Endothelial dysfunction is the basis of the physiopathological mechanisms of vascular diseases. In addition to the therapeutic activity of plant extracts, cytotoxicity is significant. This research evaluates the cytotoxicity of three vegetal extracts (*Calendulae flos* extract-CE, *Ginkgo bilobae folium* extract-GE, and *Sophorae flos* extract-SE). In vitro evaluation was performed using an endothelial cell line model (Human Pulmonary Artery Endothelial Cells—HPAEC) when a dose-dependent cytotoxic activity was observed after 72 h. The IC_50_ values were calculated for all extracts: *Calendulae flos* extract (IC_50_ = 91.36 μg/mL), *Sophorae flos* extract (IC_50_ = 68.61 μg/mL), and *Ginkgo bilobae folium* extract (IC_50_ = 13.08 μg/mL). Therefore, at the level of HPAEC cells, the cytotoxicity of the extracts follows the order GE > SE > CE. The apoptotic mechanism implied in cell death was predicted for several phytocompounds using the PASS algorithm and molecular docking simulations, highlighting potential interactions with caspases-3 and -8. In vivo analysis was performed through brine shrimp lethality assay (BSLA) when lethal, behavioral, and cytological effects were evaluated on *Artemia salina* larvae. The viability examined after 24 h (assessment of lethal effects) follows the same sequence: CE > SE > GE. In addition, the predicted cell permeability was observed mainly for GE constituents through in silico studies. However, the extracts can be considered nontoxic according to Clarckson’s criteria because no BSL% was registered at 1200 µg/mL. The obtained data reveal that all three extracts are safe for human use and suitable for incorporation in further pharmaceutical formulations.

## 1. Introduction

The vascular endothelium represents the first barrier of all elements circulating in the bloodstream (cells, pathogens, molecules) lining the lumen of the blood vessels. Cells differ morphologically, physiologically, and phenotypically along the course of the vascular bed, between arteries and veins, arterioles and venules, and even between capillaries of the same vascular bed or between different organs. However, endothelial cell populations have common functions (secretory, synthetic, immunological, metabolic) that constantly adapt to regional needs [1,2].

Under physiological conditions, endothelium provides a non-thrombogenic layer that maintains blood fluidity due to its anticoagulant and antiplatelet activity. Usually, endothelial cells limit clot formation only in the areas where it is necessary to restore vascular integrity after injury. The endothelium is involved in platelet aggregation through the von Willebrand factor (vWF), which is stored in endothelial granules (Weibel–Palade bodies) [2]. Its release triggers platelet aggregation with clot formation. At the same time, endothelial cells modulate the vascular tone by secreting vasodilators (NO, PGI2) or vasoconstrictors (angiotensin, endothelin-1, endothelin-2) and have implications in regulating the growth and homeostasis of the adjacent layer of smooth muscle cells. The activation of the growth factor receptors expressed on the surface of vascular endothelial cells (VEGFR) initiates the process of angiogenesis to adapt to pathological conditions (e.g., the formation of new vessels to maintain the supply of oxygen and nutrients) [2]. Endothelial metabolic imbalance involves the release of pro-inflammatory mediators and matrix-metalloproteinases (increasing pro-inflammatory status), platelet adhesion, and leukocyte migration to the endothelium (the initial phase of the leukocyte extravasation process in inflamed sites) leading to platelet–leukocyte interactions, aggregative processes and, as a final step, to vascular occlusion (triggering high procoagulant status), the release of large amounts of superoxide anion as a precursor of reactive oxygen species (favoring the prooxidant status) [3,4].

Over time, the active phytoconstituents from plant sources have been studied for their implications in vascular pathologies through their antioxidant, anti-inflammatory, and antithrombotic effects. The *Calendulae flos* extract (CE), the *Ginkgo bilobae folium* extract (GE)*,* and the *Sophorae flos* extract (SE) were previously prepared and characterized by spectrophotometric, FT-ICR-MS, UHPLC-HRMS/MS methods, and found to be rich in phytocompounds (rutin, quercetin, isorhamnetin, chlorogenic acid, ginkgolides, bilobetin, calendulosides, sophoricoside) belonging to several phytochemical classes (flavones, phenolcarboxylic acids, polyphenols) [5]. The antioxidant activity of these extracts (determined in previously published research by three in vitro methods: FRAP, ABTS, DPPH) is essential for treating chronic venous diseases such as vascular pathology with endothelial dysfunction [5]. Beyond the therapeutic activity, a marked importance for plant derivatives is their cytotoxicity, especially when aiming for inclusion in pharmaceutical products. In this context, the same three extracts were subjected to cytotoxicity assays. The investigation was performed in vitro on endothelial cell lines model and in vivo on *Artemia salina,* aiming to assess their suitability for further inclusion in nanoformulations. Furthermore, in silico studies were performed concerning the observed effects to predict the diffusion through the cell membrane, toxicological parameters, and potential molecular targets for phytochemicals specific to the three investigated plant extracts.

## 2. Materials and Methods

### 2.1. In Vitro Cytotoxicity

#### 2.1.1. Cell Line, Cell Culture

Cell cultures consisting of HPAEC cells (Primary Pulmonary Artery Endothelial Cells) purchased from ATCC (ATCC, PCS-100-022) were grown in the Basal Medium Vascular Cell Environment (ATCC, PCS-100-030) supplemented with Endothelial Cell Growth Kit-VEGF (ATCC, PCS-100-041) and a 1% Penicillin/Streptomycin antibiotic mixture (Sigma Aldrich, Saint Louis, MO, USA). Cells were maintained in the incubator at 37 °C, with 5% CO_2_. When they reached an 80% confluence, they were subcultured and used for analysis.

#### 2.1.2. Sample Solution Preparation from Plant Extracts

The three dry vegetal extracts analyzed in the current research were prepared from plant sources *Calendulae flos*, *Ginkgo bilobae folium,* and *Sophorae flos* by refluxing with ethanol (30 min, vegetal product/solvent ratio 1:10, successive two-step extraction), rotary evaporator concentration (Buchi R-215) and lyophilization (Christ Alpha) [5]. Stock solutions were prepared from these extracts by dissolving in DMSO (Sigma Aldrich, Saint Louis, MO, USA) for a 100 mg/mL concentration. Solutions of final concentrations (5 μg/mL, 10 μg/mL, 25 μg/mL, 50 μg/mL, 100 μg/mL) were prepared by successive dilutions (intermediate concentrations of 500 μg/mL) in the culture medium.

#### 2.1.3. MTT Assay

Ten thousand cells/well were seeded on 96-well plates and adhered to the plate surface in 24 h. The culture medium was removed, and the sample solutions (5 μg/mL, 10 μg/mL, 25 μg/mL, 50 μg/mL, 100 μg/mL) were added. The cells were incubated for 72 h. The Cell Proliferation Kit I (MTT) (11465007001 Roche, Sigma Aldrich*,* Saint Louis, MO, USA) was used as follows: 10 μL of the MTT solution/well was added, cells were incubated for 3–4 h, 100 μL of the solubilization solution/well was added, and the cells were incubated again for 30 min–2 h to solubilize the resulting formazan. After solubilization, the samples were analyzed spectrophotometrically using the Tecan Infinite 200 Pro reader (λ = 570 nm with reference at λ = 655 nm). Untreated cells and those exposed to pure solvent, DMSO 0.1% (equivalent to the highest concentration of DMSO in the samples), were used as controls. All experiments were performed in identical three-walled microplates. The results are presented as three independent determinations of mean ± standard deviation.

### 2.2. In Vivo Cytotoxicity

*Artemia* cysts (Artemia Brine Shrimp Eggs/Dohse, Aquaristik GmbH&Co. KG Otto-Hahn-953501 Gelsdorf, Germany) were used [6,7], and testing was performed on microplates (ARTOXKIT protocol) with a volume of 1 mL, t = 22–24 °C, pH = 7.5–8, in saline water of 5‰. Larvae were obtained in a 35‰ saline solution with continuous lighting and aeration for 20–24 h. Immediately after hatching, they were separated and transferred (10–20 specimens/well) to experimental vessels, wells (1 mL), in saline solutions of 5‰. The separation and introduction into the experimental wells were performed under a stereomicroscope. Microscopic preparations were made (magnification of 100× and 400×) to analyze the details, evaluate the developmental stages and highlight the effects on the larvae; the Visiocam 2 imaging system [8,9] stock solutions were prepared in distilled water (15,900 µg/mL for CE, 17,700 µg/mL for GE, and 16,100 µg/mL for SE), and serial dilutions produced samples. The assay was carried out in two stages, with evaluation in triplicate for each concentration. Control samples were represented by larvae in wells in saline solutions, maintained under the same conditions. Three types of effects were pursued: lethal, behavioral, and cytological. Lethal effects were assessed by counting affected organisms and determining mortality (or survival) for each concentration after exposure to the test solutions for 24 h. The effects were quantified in the first 24–48 h after hatching, and the concentrations that induce lethal effects (assessed by LC_50_) were determined. Behavioral effects involve evaluating the type of movement. Cytological effects were observed directly microscopically, without staining (larvae are transparent). Significant aspects (possible cytoplasmic accumulations/inclusions, cell adhesion in the epithelial layer, organogenesis) were monitored. In superior stages (II–III), cellular differentiation phenomena began with the appearance of appendicular growth buds, and cell lines that can be landmarks for organogenesis were configured.

### 2.3. In Silico Studies

#### 2.3.1. Ligand Preparation

Based our previous study, we established a small library of phytochemicals by selecting specific compounds identified in the three plant extracts or detected in high quantities [5]. Therefore, for further computational studies, we chose calendoflavoside, calendulosides E, F, G, H, and chlorogenic acid for the *Calendulae flos* extract, bilobalide, bilobetin, ginkgolides A, B, and C for the *Ginkgo bilobae folium* extract, and sophoricoside for the *Sophorae flos* extract. Moreover, isorhamnetin, quercetin, and rutin were also selected; these compounds were detected in high quantities in the *Sophorae flos* extract and in respectable amounts in the other two extracts. The 3D structures of the selected phytoconstituents were retrieved from the PubChem database [10], protonated according to the physiological pH (7.4), and energetically minimized with the YASARA Structure [11] using semi-empirical quantum mechanics (MOPAC) geometry optimization.

#### 2.3.2. The Prediction of Diffusion across the Cell Membrane

Since the toxicological and pharmacological activities of the three extracts could be primarily dependent on the potential of the active ingredients to interact with intracellular targets, we predicted the permeability of the selected phytochemicals across the cell membrane using the PerMM (Permeability of Molecules across Membranes [12]) web server. PerMM is a thermodynamics-based method that simulates the translocation of chemical compounds across a lipid bilayer consisting of dioleoyl phosphatidylcholine. Simulations were conducted at 298 K and physiological pH (7.4) using the “drag” optimization method. Deionization energies were also considered for ionizable compounds. Results were retrieved as cell membrane binding affinity (ΔG, kcal/mol) and permeability coefficients (logPerm) and coordinates required for generating transfer energy profiles as a function of distance from the membrane center [13]. Ligands considered permeable through the plasma membrane were selected for further computational studies.

#### 2.3.3. Prediction of Skin Permeability, Subcellular Localization and Toxicity

Considering that the three plant extracts were investigated for their potential use as topical formulations, the skin permeability of the selected phytochemicals was predicted with the pkCSM web server [14]. Furthermore, admetSAR [15] and ProTox-II [16] webservers predicted subcellular localization and toxicological profiles. The predicted toxicological properties were mitochondrial toxicity, cytotoxicity, mutagenicity, carcinogenicity, immunotoxicity, organ toxicity, eco-toxicity, and rat acute toxicity.

#### 2.3.4. Prediction of Biological Activity with PASS

PASS (Prediction of Activity Spectra for Substances) is a predictive algorithm available in both local and web versions that predicts many biological activities. Based on Level 2 Multilevel Neighborhoods of Atom descriptors (MNA) [17], PASS uses a Bayesian approach to estimate the probability for each ligand of being active (Pa) or inactive (Pi) on a specific target or outcome [18]. The SMILES codes of the selected compounds were used as input variables to screen for potential targets that could further support the results observed in the in vitro assay. Predicted targets with therapeutical implications were also taken into consideration.

#### 2.3.5. Molecular Docking Simulations

Molecular docking experiments were carried out to investigate the potential interactions between the selected compounds and predicted targets by PASS. Crystal structures of human caspase-3 (PDB ID: 1QDU [19]) and caspase-8 (PDB ID: 1F9E [20]) homodimers in complex with peptide inhibitors and human c-Myc/Max heterodimer in complex with DNA (PDB ID: 1NKP [21]) were retrieved from the RCSB PDB database [22]. The protein structures were prepared by removal of bound inhibitors and nucleic acids, correction of structural errors, protonation according to physiological pH, optimization of the hydrogen-bonding network, and energy minimization with the YASARA2 forcefield [23]. For docking with caspase-3 and -8, the docking simulations were carried out in a “blind” manner; the grid box centered around the entire protein. When docking against the Myc-Max heterodimer, the grid box was centered around the dimerization interface (25 × 25 × 25 Å). The AutoDock Vina v1.1.2 [24] algorithm within YASARA was used for the docking experiments, and 12 runs were executed for each ligand. Results were retrieved as the best binding pose of each ligand, with corresponding binding energy (ΔG, kcal/mol) and ligand efficiency (LE, ΔG/no. of heavy atoms) values. The protein–ligand interactions were analyzed using the BIOVIA Discovery Studio Visualizer (BIOVIA, Discovery Studio Visualizer, Version 17.2.0, Dassault Systèmes, 2016, San Diego, CA, USA).

### 2.4. Data Analysis

The analyses were performed in triplicate, and the results were displayed as a mean ± standard deviation (SD). The statistically significant differences *(p* < 0.05) between various experimental groups were established using the one-way ANOVA test from Microsoft 365 Excel^®^ v.2023 (Microsoft Corporation, Redmond, WA, USA), Levene’s test, Fisher’s F-test, Bartlett’s test, and *t*-test for two independent samples from XLSTAT 2023.1.4. by Lumivero (Denver, CO, USA).

The correlations between the bioactive constituents of the extracts [5] and their antioxidant activity (results published in our previous work [5] and displayed in Supplementary Materials) and cytotoxicity were determined using Principal Component Analysis performed with XLSTAT 2023.1.4. by Lumivero (Denver, CO, USA) through Pearson correlation [25,26].

The level of probability value *p* < 0.05 indicates statistically significant differences.

## 3. Results

### 3.1. In Vitro Cytotoxicity

MTT assay suggested that the analyzed extracts have a dose-dependent cytotoxic activity on HPAEC cell lines. The results expressed as a mean ± standard deviation are displayed in Table 1.

Table 1 shows that DMSO 0.1% had no significant cytotoxicity on HPAEC cells. The viability rate recorded in the case of 0.1% DMSO of 100 μg/mL was 99.63 ± 12.08%, vs. all plant extracts at the same concentration of 52.02 ± 9.44% (CE), 39.71 ± 12.33% (GE) and 46.70 ± 15.09% (SE), *p* < 0.05 (Table 1). For the CE, increase in the concentration caused a decrease in cell viability from 80.49 ± 15.03% (recorded at the lowest concentration of the extract, 5 μg/mL) to 52.02 ± 9.44% (corresponding to the highest concentration tested, 100 μg/mL), *p* < 0.05. After exposure to GE, the viability level decreased from 55.56 ± 11.08% to 39.71 ± 12.33% (*p* > 0.05). In the case of SE, the HPAEC cell viability diminished from 65.74 ± 7.48% to 46.70 ± 15.09% (*p* > 0.05). Considering IC_50_ values, the extract cytotoxicity increases in the following order: the *Calendulae flos* extract (IC_50_ = 91.36 μg/mL), the *Sophorae flos* extract (IC_50_ = 68.61 μg/mL) and the *Ginkgo bilobae folium* extract (IC_50_ = 13.08 μg/mL).

### 3.2. In Vivo Cytotoxicity

The brine shrimp lethality (BSL) values, expressed as a percent, are registered in Table 2.

The following concentrations of extracts did not influence *Artemia salina* larvae viability: 50, 100, 200, 400, 800, and 1200 µg/mL. Over these values, several concentrations with similar values were selected for each extract (Table 2). In the 1500–3400 µg/mL concentration range, CE has no lethal effects on *A. salina* larvae (Table 2). Substantially increased BSL% values were observed at GE (1770 and 2800 µg/mL): 29 ± 5.25 and 60 ± 10.70, *p* < 0.05. In the same concentration ranges, SE activity did not record significant differences between 1610 and 3300 µg/mL (9 ± 1.38 and, respectively, 11 ± 2.83%, *p* > 0.05). Then, we tested an additional SE concentration over 3500 µg/mL. However, the BSL% value did not increase significantly: 14 ± 4.1 vs 11 ± 2.83%, *p* > 0.05 (Table 2).

**Figure 1:**
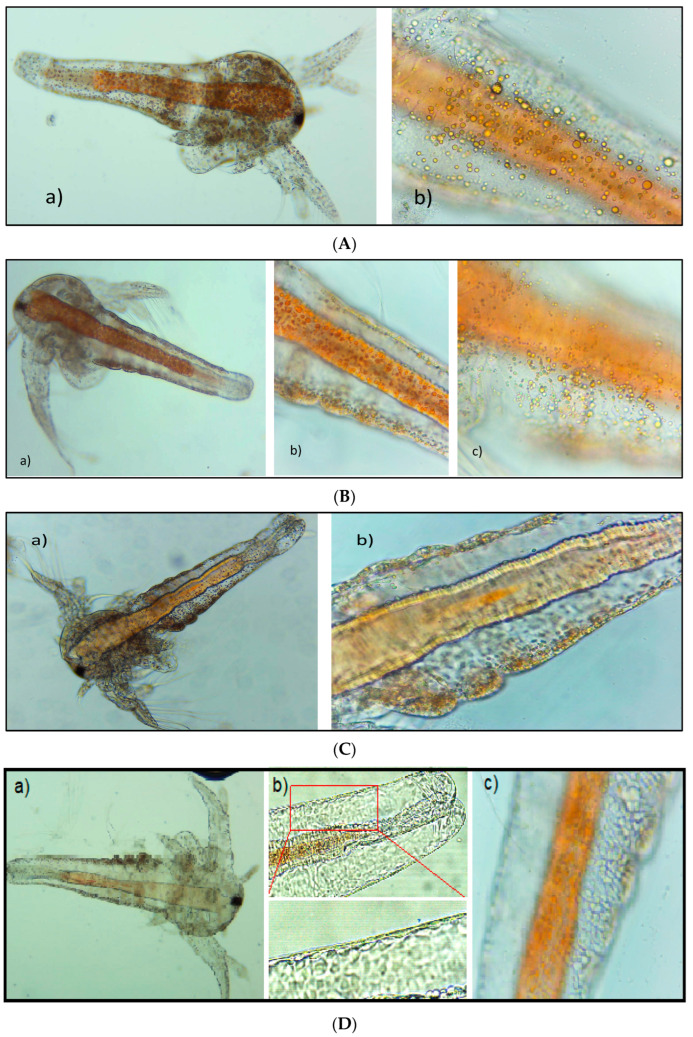
Morphological changes. (**A**) Larvae exposed to *Calendulae flos* extract after 24 h (concentrations of 2500 μg/mL): (**a**) Stage III larvae (100×); (**b**) Epithelial cells with inclusions (400×); (**B**) Larvae exposed to *Ginkgo bilobae folium* extract after 24 h (concentrations of 2800 μg/mL): (**a**) Stage III larvae (100×); (**b**) Appendicular growth area (400×); (**c**) Epithelium with inclusions (400×); (**C**) Larvae exposed to *Sophorae flos* extract after 24 h (concentrations of 5500 μg/mL): (**a**) Stage III larvae (100×); (**b**) Appendicular growth area (400×); (**D**) Control larvae: (**a**) Stage III larvae (100×); (**b**) Detail of monolayer epithelium (400×); (**c**) Detail of appendicular growth area (400×).

Several observations concerning morphological aspects are available (Figure 1). Thus, *Artemia salina* larvae exposed to the *Calendulae flos* extract (2500 µg/mL) showed large cellular inclusions without other changes (Figure 1A). For the *Ginkgo bilobae folium* extract (2800 μg/mL), after 24 h, no noticeable morphological changes were registered, and the appendicular growth zone was slowly developed (Figure 1B(a)). However, multiple large cell inclusions were observed (Figure 1B(b,c)). The *Sophorae flos* extract (5500 μg/mL) induced body deformations and cuticle adhesion loss (Figure 1C). All these morphological changes were detected by comparison with control nauplii (Figure 1D).

Regarding the behavioral effects, larvae exposed to the *Ginkgo bilobae folium* extract (2800 μg/mL, 1770 μg/mL, 1200 μg/mL, 800 μg/mL) showed apparent changes in swimming after 24 h characterized by very short, abnormal movements remaining on the bottom of the vessel. For the *Sophorae flos* extract, the larvae showed intermittent, convulsive movements, the contraction being affected.

### 3.3. Principal Component Analysis

**Figure 2:**
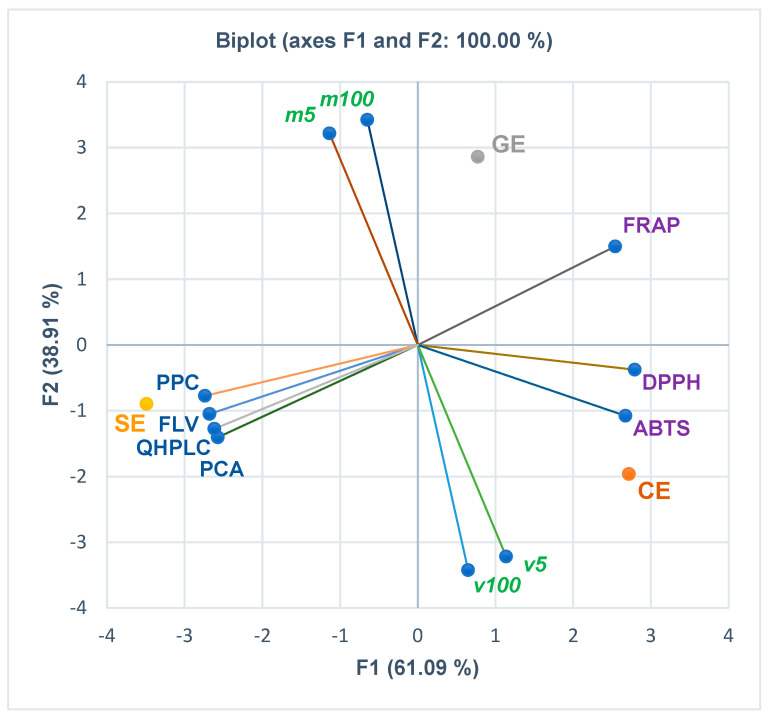
The correlations between principal classes of bioactive constituents, antioxidant effects, and in vitro cytotoxicity of all extracts. CE—*Calendulae flos* extract, GE—*Ginkgo bilobae folium* extract, SE—*Sophorae flos* extract, PCA = phenolcarboxylic acid content (g chlorogenic acid/100 g extract); FLV = flavonoid content (g rutin/100 g extract); PPC = polyphenolic content (g tannic acid/100 g extract); QHPLC = polyphenolic content from quantitative UHPLC method (mg/g); FRAP = EC_50_ (mg/mL) from ferric reducing antioxidant power assay (mg/mL); DPPH = IC_50_ from 2,2−diphenyl−1−picryl−hydrazine assay (mg/mL); ABTS = IC_50_ from 2,20−azinobis−3−ethylbenzotiazoline−6−sulfonic acid assay (mg/mL); *v5* and *v100* = viability%, *m5* and *m100* = mortality%, corresponding to extract concentrations of 5 and 100 µg/mL determined through MTT assay.

**Figure 3:**
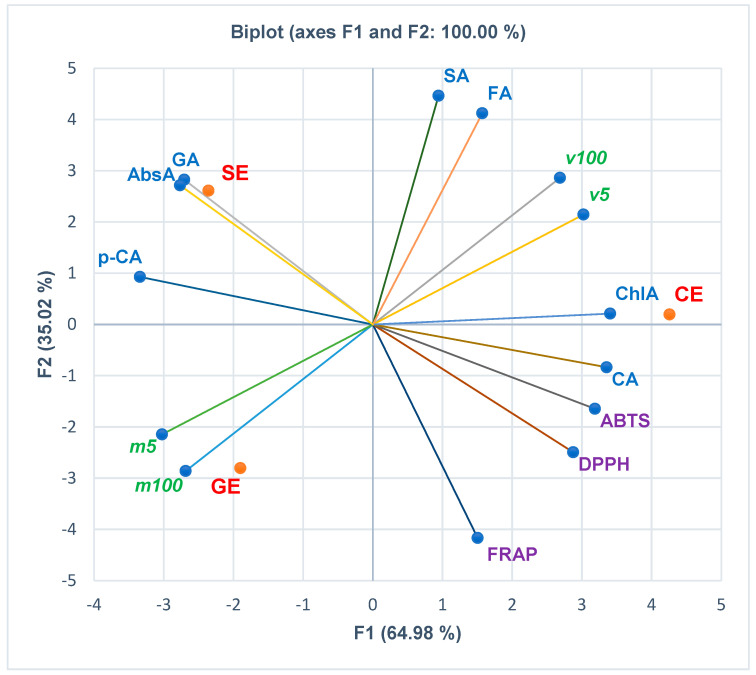
The correlations between phenolcarboxylic acids, antioxidant effects, and in vitro cytotoxicity of all extracts. CE—*Calendulae flos* extract, GE—*Ginkgo bilobae folium* extract, SE—*Sophorae flos* extract, CA = caffeic acid, *p*−CA = *p*−coumaric acid, SA = syringic acid, ChlA = chlorogenic acid, FA = ferulic acid, GA = gallic acid, AbsA = abscisic acid, FRAP = EC_50_ from ferric reducing antioxidant power assay (mg/mL); DPPH = IC_50_ from 2,2−diphenyl−1−picryl-hydrazine assay (mg/mL); ABTS = IC_50_ from 2,20−azinobis−3−ethylbenzotiazoline−6−sulfonic acid assay (mg/mL); *v5* and *v100* = viability%, *m5* and *m100* = mortality%, corresponding to extract concentrations of 5 and 100 µg/mL, determined through MTT assay.

**Figure 4:**
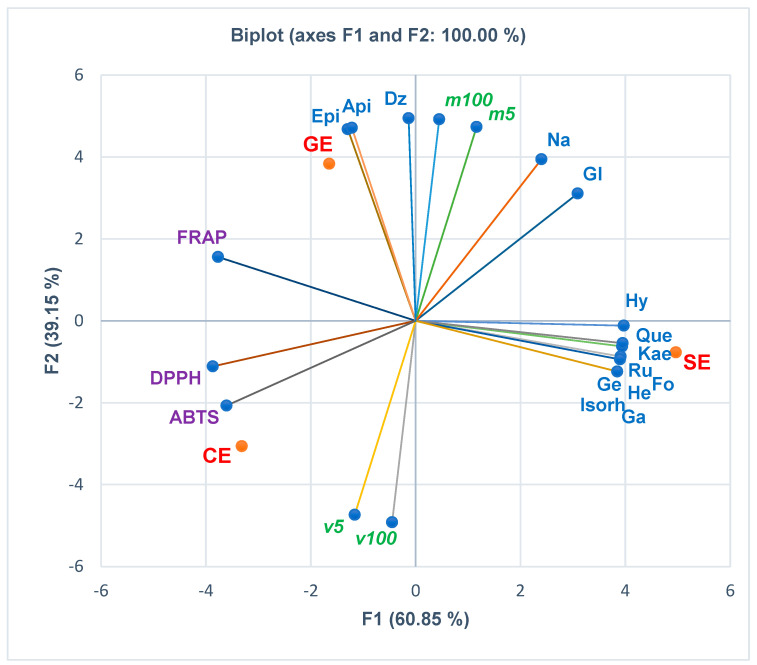
The correlations between flavonoids, antioxidant effects, and in vitro cytotoxicity of all extracts. CE—*Calendulae flos* extract, GE—*Ginkgo bilobae folium* extract, SE—*Sophorae flos* extract, Epi = epicatechin, Ge = genistin, Gl = glycitein, Hy = hyperoside, Api = apigenin, Ru = rutin, Fo = formononetin, Ga = galangin, Kae = kaempferol, He = hesperetin, Na = naringenin, Que = quercetin, Isorh = isorhamnetin, Dz = daidzein. FRAP = EC_50_ from ferric reducing antioxidant power assay (mg/mL); DPPH = IC_50_ from 2,2−diphenyl−1−picryl−hydrazine assay (mg/mL); ABTS = IC_50_ from 2,20−azinobis−3−ethylbenzotiazoline−6−sulfonic acid assay (mg/mL); *v5* and *v100* = viability%, *m5* and *m100* = mortality%, corresponding to extract concentrations of 5 and 100 µg/mL determined through MTT assay.

**Figure 5:**
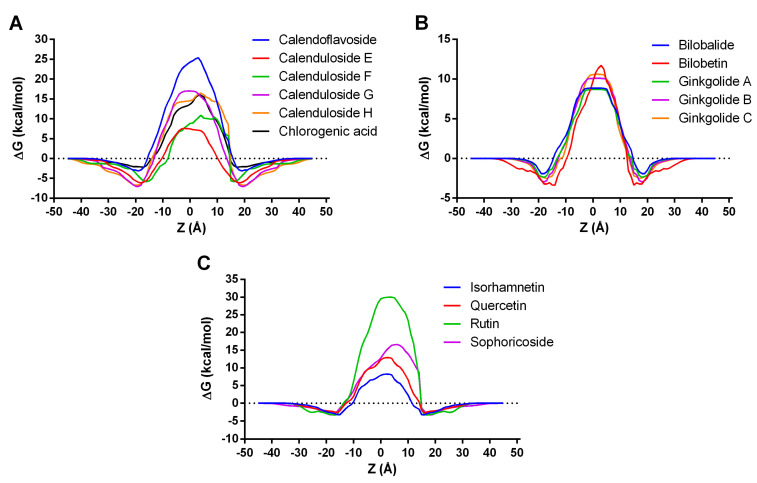
Variation of transfer energy as a function of distance from the center of the lipid bilayer (Z). (**A**) Transfer energy profiles for compounds detected in *Calendulae flos* extract; (**B**) Transfer energy profiles for compounds detected in *Ginkgo bilobae folium* extract; (**C**) Transfer energy profiles for compounds detected in *Sophorae flos* extract.

The correlations between the phenolcarboxylic acids, flavones, and polyphenols, antioxidant activities (assessed by the three methods FRAP, DPPH, and ABTS), and in vitro cytotoxicity are displayed in Figure 2, Figure 3, Figure 4 and Figure 5. At the same time, the biplots from all the previously mentioned figures indicate the place of each extract reported to these correlations.

Figure 2 and the Correlation Matrix from Supplementary Materials show that viability levels at extract concentrations of 5 µg/mL and 100 µg/mL *(v5* and *v100)* are moderately correlated with the antioxidant activity of the extracts expressed as ABTS IC_50_ (*r* = 0.666, *r* = 0.518, *p* > 0.05). Moreover, *v5* moderately correlates with DPPH IC_50_ (*r* = 0.500, *p* > 0.05), while *v100* shows a low positive correlation with DPPH IC_50_ and PCA, QHPLC, and FLV (*r* = 0.334, *r* = 0.177, *r* = 0.137, *r* = 0.069, *p* > 0.05). Lethality levels (*m5* and *m100*) register a low positive correlation with FRAP IC_50_ (*r* = 0.023, *r* = 0.205, *p* > 0.05) and PPC (*r* = 0.012, *r* = 0.194, *p* > 0.05). Furthermore, *m5* is poorly correlated with PPC, FLV, QHPLC, and PCA (*r* = 0.194, *r* = 0.114, *r* = 0.046, *r* = 0.006, *p* > 0.05). DPPH shows a strong correlation with ABTS and FRAP (*r* = 0.979, *r* = 0.854, *p* > 0.05); ABTS and FRAP record a lower one (*r* = 0.731, *p* > 0.05). Figure 2 and Correlation Matrix from Supplementary Materials reveal that all total phenolic contents show an appreciable negative correlation with antioxidant activity (*r* = −0.750–−0.991, *p* > 0.05). PCA and QHPLC highlight a statistically significant negative correlation with FRAP (*r* = 0.999, *r* = 0.998, *p* < 0.05).

Figure 3 displays the correlations between phenolcarboxylic acids, antioxidant effects, and in vitro cytotoxicity of the extracts. Hence, Figure 3 and the Correlation Matrix from Supplementary Materials show that viability levels at both extract concentrations (5 and 100 µg/mL) are considerably positively correlated with a large part of phenolcarboxylic acids: ferulic acid (*r* = 0.819, *r* = 0.910, *p* > 0.05), chlorogenic acid (*r* = 0.907, *r* = 0.815, *p* > 0.05), syringic acid (*r* = 0.689, *r* = 0.810, *p* > 0.05) and caffeic acid (*r* = 0.789, *r* = 0.664, *p* > 0.05). Mortality levels (*m5* and *m100*) show good and moderate positive correlations with *p*-cinnamic acid (*r* = 0.777 and *r* = 0.649, *p* > 0.05) and a low correlation with gallic acid (*r* = 0.423, *r* = 0.250, *p* > 0.05) and abscisic acid (*r* = 0.449, *r* = 0.279, *p* > 0.05). The HPAEC cell viability (%) correlates positively with DPPH and ABTS, while mortality (%) correlates with FRAP. Caffeic acid and syringic acid are highly positively correlated with DPPH and ABTS (*r* = 0.818–0.984, *p* > 0.05) and moderately correlated with FRAP (*r* = 0.596, *r* = 0.400, *p* > 0.05). Abscisic acid shows a significant statistically negative correlation with DPPH (*r* = −0.998, *p* < 0.05) and considerable correlations with ABTS and FRAP (*r* = −0.966, *r* = −0.883, *p* > 0.05). All the other phenolcarboxylic acids report negative correlations (strong to low) with all antioxidant activities (Figure 3 and Correlation Matrix from Supplementary Materials); only ferulic acid has a minimal positive correlation with ABTS (*r* = 0.116, *p* > 0.05).

Figure 4 displays the correlations of flavonoids with in vitro cytotoxicity and antioxidant effects of plant extracts. The HPAEC cell mortality (%) at the lowest extract concentration (5 µg/mL) positively correlates with all flavonoids. Both *m5* and *m100* recorded a substantial positive correlation with daidzein (*r* = 0.949, *r* = 0.989, *p* > 0.05), naringenin (*r* = 0.939, *r* = 0.860, *p* > 0.05), apigenin (*r* = 0.821, *r* = 0.911, *p* > 0.05), epicatechin (*r* = 0.809, *r* = 0.902, *p* > 0.05), and glycitein (*r* = 0.828, *r* = 0.712, *p* > 0.05) and a low correlation with hyperozide and quercetin (*r* = 0.270–0.135, *r* = 0.185–0.003, *p* > 0.05). The endothelial cell viability at the highest extract concentration (100 µg/mL) shows a minimal positive correlation with genistein, rutin, formonetin, galangin, kaempferol, hesperidin, and isorhamnetin (*r* = 0.013–0.139, *p* > 0.05). Moreover, most flavonoids report a negative correlation with FRAP and DPPH. ABTS negatively correlates with all flavonoids. Isorhamnetin has a significant negative correlation with FRAP (*r* = −0.998, *p* < 0.05). Epicatechin and apigenin correlate moderately with FRAP (*r* = 0.607, *r* = 0.590, *p* > 0.05) and minimally with DPPH (*r* = 0.105, *r* = 0.085, *p* > 0.05). At the same time, daidzein shows a low correlation with FRAP (*r* = 0.347, *p* > 0.05, Figure 4 and Supplementary Materials).

### 3.4. Computational Studies

#### 3.4.1. Permeability across Cell Membrane Prediction

Several previously assessed phytochemicals contained by the investigated plant extracts were subjected to thermodynamics simulation to predict the translocation through the cell membrane using the PerMM server since internalization in the intracellular space is required prior to interacting with certain molecular targets. The predicted free energy of binding to the plasma membrane constituents and calculated permeability coefficients are shown in Table 3. The lowest binding energies were predicted for calendulosides, while the highest value was observed for bilobalide. The permeability coefficients varied from −18.13 (rutin) to −6.89 (calenduloside E), with higher values corresponding to a better diffusion. Permeability coefficients lower than −10 were predicted for calendoflavoside, calenduloside G, calenduloside H, chlorogenic acid, rutin, and sophoricoside, which we considered to be highly unlikely to permeate through the cell membrane due to either high molecular weight or high polarity. Therefore, we selected bilobalide, bilobetin, ginkgolides A, B, and C, isorhamnetin, and quercetin as the phytoconstituents with the highest probability of diffusing through the cell membrane and exerting biological effects.

The membrane transfer energy profiles along the lipid bilayer normal (Z) for the investigated compounds are shown in Figure 5. Among the constituents detected in the *Calendulae flos* extract (Figure 5A), calendulosides E and F required the lowest transfer energy for membrane diffusion, the transfer energy profile of calenduloside E being more symmetric than that of calenduloside F. All the phytochemicals specific to the *Ginkgo bilobae folium* extract had similar transfer energy profiles, bilobalide, and ginkgolide A requiring the lowest transfer energies (Figure 5B). Rutin, previously found in high concentrations in the *Sophorae flos* extract, required a markedly higher transfer energy when compared to quercetin, isorhamnetin, and sophoricoside (Figure 5C). Nonetheless, isorhamnetin required the least amount of transfer energy for crossing the hydrophobic portion of the membrane, followed by quercetin.

#### 3.4.2. Skin Permeability, Subcellular Localization, and Toxicity Prediction

Further, several parameters were predicted for the highly likely compounds to translocate across the cell membrane (Table 4). All phytochemicals had comparable values for the predicted logarithmic skin permeability coefficient (log Kp), ranging from −2.762 to −2.735, falling within the acceptable range for druglike compounds [27]. All compounds were predicted to localize in the mitochondrion following cell membrane diffusion and exert mitochondrial toxicity, which could explain the observed cytotoxic effects in HPAEC cells to a certain extent. On the other hand, all the compounds were predicted as non-cytotoxic, which implies that other uninvestigated phytoconstituents could be partly responsible for cytotoxicity. Moreover, only bilobalide and quercetin were predicted as mutagenic, albeit with very low probabilities (0.50 and 0.51). Quercetin was also predicted as carcinogenic, with a probability of 0.68. All the assessed compounds were predicted as toxic to immune cells, the probabilities ranging from 0.55 (bilobalide) to 0.99 (calendulosides E and F).

No phytochemicals were predicted as hepatotoxic at the organ level, while bilobalide, bilobetin, and ginkgolides were considered nephrotoxic. Furthermore, bilobalide, ginkgolides, and quercetin were positive for toxicity in the respiratory system (0.53–0.62 probabilities), while only ginkgolide C was negative for reproductive toxicity. The highest rat acute toxicity (LD_50_) was predicted for bilobalide (90 mg/kg), followed by quercetin (159 mg/kg), ginkgolides (500 mg/kg), calenduloside E (1750 mg/kg), bilobetin (4000 mg/kg) and isorhamnetin (5000 mg/kg). For calenduloside F, the acute toxicity could not be determined.

Concerning eco-toxicity, all compounds were predicted as safe for honeybees. Moreover, bilobalide and ginkgolide C were predicted to be toxic to Crustacea, such as *Daphnia magna*, while only ginkgolides B and C were predicted as safe to fish species.

#### 3.4.3. PASS Activity Prediction

The PASS algorithm predicted the screened compounds’ potential molecular targets and biological activities (Table 5). The software yielded 708 potential targets or activities for bilobalide, 878 for bilobetin, 411 for calenduloside E, 409 for calenduloside F, 138 for ginkgolide A, 126 for ginkgolide B and C, 933 for isorhamnetin and 1429 for quercetin. By analyzing the probabilities of being active, especially for the activities with probabilities higher than 0.5, we observed that bilobetin, calendulosides, isorhamnetin, and quercetin had probabilities higher than 0.8 to act as apoptosis inducers at the cellular level (Table 5). Thus, the cytotoxicity of the *Ginkgo bilobae folium* extract could be partly attributed to bilobetin. Interestingly, calendulosides had very high probabilities (close to 1) to act as caspase-3 inducers, followed by isorhamnetin (0.656), bilobetin (0.649), quercetin (0.499) and bilobalide (0.257). For bilobalide, the probability of being active as a caspase-3 inducer was comparable to that of being inactive (0.222). Furthermore, calendulosides E and F had remarkably high probabilities of acting as caspase-8 inducers, while bilobalide, bilobetin, isorhamnetin, and quercetin had probabilities of being active on caspase-8 lower than 0.5. Therefore, bilobalide, bilobetin, isorhamnetin, quercetin, and especially calendulosides could induce apoptosis by enhancing the activity of caspase-3 and caspase-8.

Another interesting outcome was the prediction of the screened compounds as potential inhibitors of transcription factor c-Myc. The highest probability of exerting the c-Myc inhibitory activity was predicted for phytoconstituents from the *Calendulae flos* and *Ginkgo bilobae folium* extracts. This specific molecular mechanism would not explain the cytotoxic effect in HPAEC cells but could be harnessed for therapeutical applications since c-Myc activity promotes apoptosis in healthy cells [28]. Other notable predicted activities were vasoprotective, capillary fragility treatment, antineoplastic, antimetastatic, cytokine release inhibitor, antioxidant, antithrombotic, proliferative disease treatment, and vascular dementia treatment.

#### 3.4.4. Molecular Docking Studies on Caspase-3, Caspase-8 and c-Myc

Lastly, molecular docking experiments were performed to evaluate the potential of the selected phytoconstituents to bind to the targets predicted with PASS (Table 6). Predicted binding energies for caspase-3 ranged from −11.182 kcal/mol (calenduloside E) to −7.399 kcal/mol (bilobalide), while the highest ligand efficiencies were observed for isorhamnetin, quercetin, and bilobalide (Table 6). For caspase-8, binding energies varied from −10.467 kcal/mol (calenduloside F) to −7.624 kcal/mol (quercetin), the highest ligand efficiency being noted also for quercetin, bilobalide, and isorhamnetin. Interestingly, bilobalide, bilobetin, calenduloside E, isorhamnetin, and quercetin were docked into the dimerization site of caspase-3. At the same time, the other ligands were predicted to bind to the catalytic site, thus probably acting as potential inhibitors rather than enhancers. Furthermore, bilobalide, bilobetin, calenduloside F, ginkgolide C, isorhamnetin, and quercetin were predicted to bind to the dimerization site of caspase-8, thus strengthening the interaction between the two monomers.

After docking into the c-Myc/Max dimerization site involved in the binding of inhibitors, the predicted affinities varied between −7.134 kcal/mol (bilobetin) and −5.816 kcal/mol (quercetin). Higher ligand efficiency values were obtained for bilobalide, quercetin, isorhamnetin, and ginkgolides. However, quercetin and isorhamnetin were predicted to bind into an adjacent site, less likely to be involved in disrupting the interactions between c-Myc and Max.

**Figure 6:**
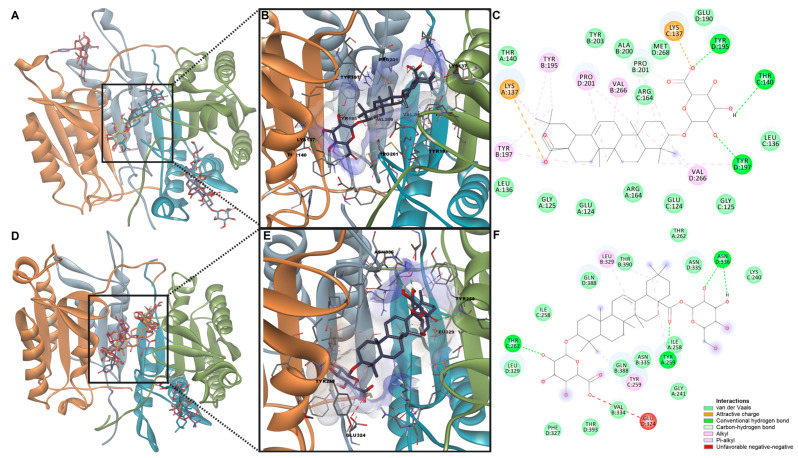
(**A**) Predicted poses of screened ligands after docking with caspase-3 dimer. (**B**) Predicted protein–ligand complex between caspase-3 and calenduloside E. (**C**) 2D interaction diagram between caspase-3 and calenduloside E. (**D**) Predicted poses of screened ligands after docking with caspase-8 dimer. (**E**) Predicted protein–ligand complex between caspase-8 and calenduloside F. (**F**) 2D interaction diagram between caspase-3 and calenduloside F.

We further highlighted the predicted interactions between calenduloside E and caspase-3 and between calenduloside F and caspase-8, respectively (Figure 6). Judging by the predictions, we could assume that calenduloside E could be selective towards caspase-3, while calenduloside F might preferably bind to caspase-8. The simulations showed both compounds to potentially enhance the stabilization of the dimeric structures by being involved in several favorable contacts with amino acid residues within both monomers. For instance, calenduloside E interacted with lysines 137 from both monomers through attractive charges. The protein ligand complex is further stabilized by hydrogen bonds with Tyr197, Tyr195, and Thr140 from one monomer, while interactions with the other monomer are mostly hydrophobic (Figure 6C). Calenduloside F interacted with caspase-8 homodimer by engaging in hydrogen bonds with Thr262m Tyr259 and Asn336 and in nonpolar alkyl and pi-alkyl interactions with Tyr259 and Leu329. However, the ionized carboxylic moiety and Glu324 form an unfavorable negative–negative contact.

**Figure 7:**
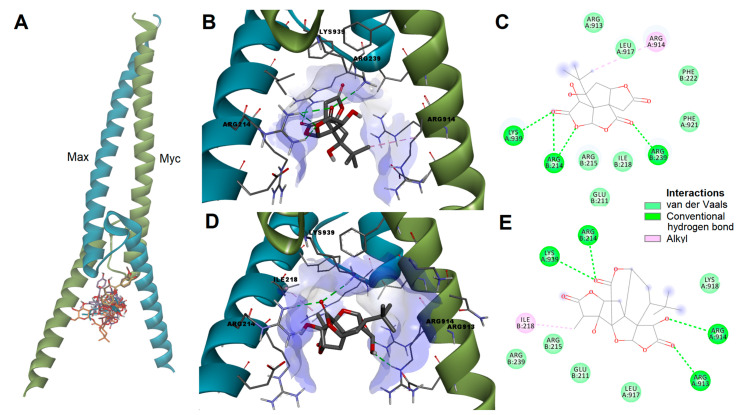
(**A**) Predicted poses of screened ligands after docking into c-Myc/Max dimerization interface. (**B**) Predicted protein–ligand complex between bilobalide and c-Myc/Max complex. (**C**) 2D interactions diagram between bilobalide and c-Myc/Max complex. (**D**) Predicted protein–ligand complex between ginkgolide A and c-Myc/Max complex. (**E**) 2D interactions diagram between ginkgolide A and c-Myc/Max complex.

Bilobalide and ginkgolide A were among the screened compounds with the highest probability of inhibiting c-Myc and with the most favorable docking results. Bilobalide could potentially disrupt the dimerization between c-Myc and Max by forming a hydrogen bond with key residue Lys939 and a hydrophobic interaction with Arg914 within c-Myc, and by engaging in hydrogen bonding with Arg214 and Arg239 within Max, thus preventing the stabilization of the heterodimer (Figure 7B,C). Ginkgolide C could bind to the dimerization interface by forming hydrogen bonds with residues Arg913, Arg914, and Lys939 within c-Myc and Arg214 within Max while also forming several hydrophobic contacts such as alkyl interactions with Ile218 and van der Waals interactions (Figure 7D,E).

## 4. Discussion

Current work evaluated the cytotoxicity of three extracts (*Calendulae flos* extract, *Ginkgo bilobae folium* extract, *Sophorae flos* extract) in vitro on endothelial cell lines (HPAEC) and in vivo on *Artemia salina*. These three dry extracts were obtained (ethanol reflux extraction, rotary evaporator concentration, lyophilization) and phytochemically characterized (spectrophotometric, FT-ICR-MS, UHPLC-HRMS/MS) in our previously published research [5]. By quantitative UHPLC-HRMS/MS, we found that CE is rich in chlorogenic acid (20,676.63 μg/g extract), isorhamnetin (11,286.93 μg/g extract), and rutin (2165.42 μg/g extract), GE has a significant content of isorhamnetin (5032.60 μg/g extract), quercetin (4504.66 μg/g extract), and rutin (3907.47 μg/g extract). SE is known for its rutin (104,186.77 μg/g extract), isorhamnetin (97,049.32 μg/g extract), and quercetin (46,678.34 μg/g extract) content [5]. The extracts also contain characteristic compounds such as calendulosides (E, E/G, H) for CE, ginkgolides (A, B, C), bilobetin, bilobalide for GE, and sophoricoside for SE [5]. These three extracts were chosen based on their phytochemical composition. SE is notable for its flavonoid content, known for antioxidant and anti-inflammatory effects, CE also contains calendulosides, known for their role in re-epithelialization, and GE, in addition to flavonoids, contains ginkgolides known for their anti-aggregant effect [5]. Each of the three extracts can be useful for endothelial disfunction pathologies depending on the stage of the disease.

Regarding cytotoxicity results on HPAEC endothelial cell lines, interpreted comparatively, they suggest that the most cytotoxic was the *Ginkgo bilobae folium* extract, and the least cytotoxic was the *Calendulae flos* extract, with the *Sophorae flos* extract showing intermediate cytotoxicity.

Some phytoconstituents were tested on different endothelial cell lines evaluating both activity and cytotoxicity. A study evaluated the activity of calenduloside E on an ischemia/reperfusion cell model (OGD/R, oxygen–glucose deprivation/reperfusion type) on HT22 cell lines; at the same time, it was observed that at concentrations below 16 μg/mL, no cytotoxic effects were recorded on these cell lines [29]. Ginkgolide B did not affect the survival of b.End3 cells and HUVECs. Ginkgolide B promoted the proliferation of the two cell lines in a dose-dependent manner. At the same time, when increasing the dose of ginkgolide B, the favoring of repair processes after cell damage (scratch healing) was observed. The results also indicated the induction of angiogenesis on both b.END2 cells and HUVECs [30]. Sophoricoside was tested for its antiallergic effect in asthma on human mast cell (HMC-1) cell lines; it reduced the amount of prostaglandins and leukotrienes in HMC-1 cells without affecting cell viability [31].

Other studies were conducted to find beneficial effects on various endothelial cell lines. Calenduloside E proved an antiapoptotic activity on endothelial cell lines (HUVEC—human umbilical vein endothelial cells) [32]. Chlorogenic acid increased cell viability and NO production, and reduced S-nitrosothiols, nitrite, and nitroso species in the human aortic endothelial cell (HAEC) model [33,34]. The *Ginkgo* extract favors thrombomodulin (TM) expression and t-PA secretion contributing to the protection of the endothelium in the procoagulant and prothrombotic phases (observations by assay on HUVEC cells) [35]. Ginkgolide A demonstrated antiapoptotic effects by modulating the expression of miR-224 and regulating the expression of p21 at the endothelial level (PMVEC—pulmonary microvascular endothelial cells) [36]. Ginkgolide B has an anti-inflammatory effect observed on HUVEC cells by downregulating LOX-1 expression. At the same time, it inhibits the expression of MCP-1, ICAM-1, VCAM-1, and leukocyte adhesion at the level of HUVEC endothelial cells [37,38]. Rutin induces NO production in endothelial cells (HUVEC) by inducing eNOS gene expression, eNOS protein synthesis, and eNOS activity. Another effect of rutin observed on HUVEC endothelial lines is the promotion of the fibroblast growth factor (bFGF) expression [39]. Quercetin proved protective over endothelial cells (the HBMEC model—human brain microvascular endothelial cell) by preventing damage to the mitochondrial membrane and inhibiting apoptosis [40]. Other polyphenolic compounds have shown a protective effect on endothelial cells (HPAEC—human pulmonary artery endothelial cells) by increasing the level of NO and decreasing the level of endothelin 1, restoring the balance of vasoactive substances [41].

In addition to these beneficial activities at the endothelial level, it is essential to know the cytotoxic effect. Most of the compounds found in these three extracts belong to the class of flavonoids. Other studies have examined the cytotoxicity of some flavonoids (including apigenin, 3-hydroxyflavone, kaempferol, luteolin, naringenin, quercetin, rutin) on human cell lines: TIG-1 (human embryonic lung fibroblasts) and HUVE (umbilical vein endothelial cells) [42]. The level of intracellular ROS was measured (in the case of TIG-1 cells considered standard cell lines) to determine the cytotoxic mechanism. The ROS production was increased in the presence of apigenin, luteolin, 3-hydroxyflavone, quercetin, and kaempferol [42]. For HUVE cells, luteolin, quercetin, and 3-hydroxyflavone had high toxicity; naringenin, apigenin, and kaempferol were significantly toxic, and rutin was nontoxic. The study suggests that flavonoids exert cytotoxic action by increasing intracellular ROS levels. At high concentrations, flavonoids become prooxidants [42]. In vitro testing on endothelial cell lines (HPAEC) revelaed that the cytotoxicity of the analyzed extracts was dose-dependent; it could be explained based on the increase in the concentration of flavonoids brought into contact with the cells. Also, the effect of flavonoids can be influenced by the presence of other compounds in the cellular environment or by the type of cells on which the determination is made. Our Principal Component Analysis shows that the HPAEC cell mortality (%) at the lowest extract concentration (5 µg/mL) positively correlates with all flavonoids. The mortality rate at both extracts concentrations (5 and 100 µg/mL) recorded a substantial positive correlation with daidzein, naringenin, apigenin, epicatechin, and glycitein, and a low positive correlation with hyperozide and quercetin. At the same time, the Pearson correlation shows that viability levels at both extract concentrations (5 and 100 µg/mL) are positively correlated with a large part of phenolcarboxylic acids: ferulic acid, chlorogenic acid, syringic acid, and caffeic acid. Concomitantly, both mortality levels (for the lowest and highest extract concentration) reported good and moderate positive correlations with *p*-cinnamic acid and low correlations with gallic acid and abscisic acid.

Most cytotoxicity studies have been conducted for the development of anticancer drugs. The mechanisms are not fully elucidated; in addition to the possibility of a prooxidant effect, other potential mechanisms have been proposed that could also apply to endothelial cells: induction of apoptosis, inhibition of cell proliferation decreasing cell viability, inhibition of angiogenesis. A previous study analyzed the cytotoxicity of the *Ginkgo biloba* ethanolic extract (70:30 *v*/*v*) on liver carcinoma cells (HepG2) and normal liver cells (THLE-2) when it inhibited cell colony formation in a dose-dependent manner [43]. Another study evaluated the cytotoxicity of the methanolic extract of *Ginkgo bilobae* on cancerous (A2058, HCT116) and non-cancerous cell lines (McCoy-Plovdiv cells), and it was found to affect the viability of tumor cell lines in a dose- and time-dependent manner, while for normal cells, it favored proliferation [44]. The *Calendulae flos* ethanolic extract’s cytotoxicity was evaluated on non-cancerous human keratinocyte cell lines (HaCaT), proving to be nontoxic at concentrations of 5% at 4 h of exposure. However, they had significant toxicity after 48 h of exposure. At lower concentrations (1%), the extracts were nontoxic upon exposure for 24 h. The extracts could contain toxic substances, but in small quantities and with low permeability through the cell membrane. After a certain contact period with cells, these components could concentrate in cells and reduce viability, possibly through a prooxidant mechanism [45]. Similar results were recorded for *Calendula* extracts on human fibroblasts (HSF); the inhibition of mitochondrial dehydrogenase was found as a mechanism of cytotoxicity, but this occurred after the destruction of the cell membrane. It can be concluded bidirectionally that both the cell membranes are sensitive to the action of the compounds in the extract. They both act on the membrane and the mitochondrial enzyme; the inhibition of the mitochondrial enzyme activity can result from the destruction of the cell membrane without the substances acting directly on the enzyme [46]. Proapoptotic and antiapoptotic effects differ depending on cell type. For extracts from *Sophorae flos*, apoptotic effect on cancer cells and antiapoptotic effect on normal HaCaT cells [47,48] were demonstrated.

In vivo*,* the cytotoxicity assessment was conducted using the BSLA (brine shrimp lethality assay) method, a preliminary test. The species is *Artemia salina* (*Artemiidae* Family), a primitive aquatic arthropod in Romanian salty lakes (e.g., Techirghiol Lake, Brăila Salt Lake). *Artemia salina* larvae in the first stage of development are used for cytotoxicity tests because they consume their yolk sac reserves in the first 48 h, and the digestive epithelium is poorly developed. It does not allow exchanges with the external environment. The digestive tract is closed, and the exchanges are made only at the level of the membrane, with the help of epithelial cells [49,50,51].

The use of *Artemia salina* as a biotester is based on the following considerations: short life cycle, easy obtention of larvae, small size, larvae in the naupliar stage I (in the first 24 h after hatching) allow the evaluation of small amounts of test substance. The evaluation of substances is based on membrane penetration mechanisms, not interfering with digestion, transparent (allow cytological evaluation in vivo with or without dyes) [52,53]. Other advantages are represented by the economic method (uses a small amount of test material and no special conditions are required), rapidity, repeatability (following the ARC—Artemia Reference Center test) [50]; in addition, it is an in vivo model widely used for plant extracts, and it is standardized and effectively replaces other test methods. There are limitations to the use; for example, an attempt was made to evaluate the antioxidant activity of rutin on the *Artemia salina* model when it was found that rutin in high concentrations kills crustaceans and prevents determination [54].

According to the literature data, the effects of *Artemia* larvae exposed to solutions of plant extracts are classified as follows: extracts with LC_50_ over 1000 µg/mL are nontoxic, LC_50_ of 500–1000 µg/mL have low toxicity, LC_50_ 100–500 µg/mL are of medium toxicity, while extracts with LC_50_ between 0 and 100 µg/mL are highly toxic [55]. In the case of the analyzed extracts, they were compared to the highest concentrations tested; the most substantial cytotoxic effect was recorded for the *Ginkgo bilobae folium* extract, with a lethality of 29% at a concentration of 1770 μg/mL, followed by the *Sophorae flos* extract which recorded a lethality of 9% at a concentration of 1610 μg/mL. The *Calendulae flos* extract showed no lethality at the tested concentrations. Thus, all three analyzed extracts have LC_50_ above 1000 μg/mL and can be considered nontoxic.

All plant extracts analyzed in vitro and in vivo showed similar results; the sequence describing the cytotoxic effect is GE > SE > CE. According to Clarkson’s criteria, they can be considered nontoxic and suitable for incorporation into pharmaceutical products.

In the current research, in silico studies followed predictions on cell permeability when the best results were recorded for bilobalide, bilobetin, ginkgolide (A, B, C), isorhamnetin, and quercetin. All these compounds were found in the *Ginkgo bilobae folium* extract and could explain the cytotoxicity on the tested models (HPAEC, BSLA). It is not excluded that other phytoconstituents from the extract contribute to this effect. Phenolic compounds can change their degree of dissociation depending on local pH [56]; in pathological conditions, the permeability can differ due to vascular damage. An important aspect is subcellular localization. According to the predictions, the tested compounds were located at the mitochondrial level. Moreover, all compounds (excepting calenduloside F) were predicted to possess mitochondrial toxicity, which can contribute to the observed cytotoxicity. Regarding the PASS predictions, the number of predicted molecular targets decreased in the following order: quercetin, isorhamnetin, bilobetin, bilobalide, calenduloside E, calenduloside F, ginkgolide A, ginkgolides B, and C. The potential apoptotic mechanism was especially noted for bilobetin (an inducer of caspase-3) and calendulosides E and F (inducers of caspase-3 and caspase-8, respectively).

The activity of isorhamnetin and quercetin towards caspases is not to be neglected since they have high binding efficiency. Previous studies suggested that quercetin favored mitochondrial-mediated apoptosis when it induced the expression and activity of caspase 3/7 (up-regulation of Casp3 and overexpression of cl-Casp3) as well as caspase-9 (on LBC3 and A172 cells) [56]. It also acts in a proapoptotic manner by reducing the transmembrane potential and DNA destruction (observed on T24 cells) [57]. Compared to quercetin, isorhamnetin increased the caspase-3 activity by about 2.5 times on A549 cells and 3.5 times on HCC-44 cells [58]. Isorhamnetin also increased the activity of caspase-8 (on both cell lines) and caspase-9 (on HCC-44). Thus, these compounds activate both pathways of inducing apoptosis: intrinsic (release of cytochrome c in the cytoplasm, cleavage of caspase-9 and activation of caspase-3) and extrinsic (sequential activation of caspases-8 and -3) [58]. Another mechanism for quercetin was decreasing the expression of the antiapoptotic proteins (Bcl-2, Bcl-XL) and increasing that of the proapoptotic ones (Bim, Bad, Bax) [59,60].

Other studies reported that ginkgolide B induced apoptosis through several mechanisms, including producing reactive oxygen species (which controls the JNK pathway), reducing mitochondrial membrane potential, and activating caspase-3 [61].

Myc expression at the cellular level depends on mitogenic stimuli and is necessary for proliferation, differentiation, and cell survival. It must dimerize with Max to bind to DNA and exercise its proliferative function, Myc-Max dimer being its active form [62,63]. Reduction in c-Myc expression was associated with endothelial dysfunction (development of pro-inflammatory phenotype) [64]. Docking studies were performed based on PASS results, suggesting that the tested phytocompounds could interact with the c-Myc/Max heterodimer, indicating that bilobalide and ginkgolides A and C could interfere with dimerization and inhibit c-Myc transcriptional activity.

For some phytoconstituents, we also predicted relevant probabilities of organ toxicity: nephrotoxicity (ginkgolides) and respiratory system toxicity (ginkgolides, quercetin). These results suggest the assessment of the effects of the entire phytocomplex (as extract) as a research direction in further in vivo studies.

## 5. Conclusions

The current research evaluated the cytotoxicity of three plant extracts which were previously phytochemically characterized (*Calendulae flos* extract, *Ginkgo bilobae folium* extract, and *Sophorae flos* extract). In vitro analysis was performed on the endothelial cell line model (HPAEC) when the most cytotoxic was the *Ginkgo bilobae folium* extract and the least cytotoxic was the *Calendulae flos* extract. The cytotoxic effects could be beneficial in proliferative vascular diseases. The same three extracts were subjected to in vivo cytotoxicity analysis (BSLA test), leading to the conclusion that the extracts are nontoxic according to Clarckson’s criteria. Considering that the endothelium is already affected in endothelial dysfunction pathologies, the active ingredients assumed as a treatment must not bring additional damage. Extensive in silico studies investigated the membrane permeability of several phytocompounds, which was higher for isorhamnetin, quercetin, bilobalide, bilobetin, and ginkgolides A, B, and C.

Moreover, computational studies predicted their potential localization at the mitochondrial level and cytotoxic mechanisms based on interaction with caspase-3 and caspase-8. Predictions using PASS and molecular docking revealed potential inhibitory effects on c-Myc for bilobalide and ginkgolides. Further research will focus on developing and characterizing nano-formulations based on these three extracts with potential applications in the treatment of vascular pathologies characterized by endothelial dysfunction such as chronic venous disease.

## Figures and Tables

**Table 1:** MTT Assay results after 72 h—the effects of plant extracts of various concentrations on HPAEC cell viability (%).

Sample/ Control	Concentration				
	5 μg/mL	10 μg/mL	25 μg/mL	50 μg/mL	100 μg/mL
CE	80.49 ± 15.03 ^a^	74.62 ± 13.44	65.11 ± 6.70 ^b^	53.87 ± 11.69	52.02 ± 9.44 ^a, x^
GE	55.56 ± 11.08	51.63 ± 7.73	42.43 ± 6.55 ^b^	43.07 ± 11.74	39.71 ± 12.33 ^y^
SE	65.74 ± 7.48	61.74 ± 10.09	55.86 ± 2.34	48.94 ± 10.01	46.70 ± 15.09 ^z^
DMSO 0.1%					99.63 ± 12.08 ^x, y, z^

CE—*Calendulae flos* extract, GE—*Ginkgo bilobae folium* extract, SE—*Sophorae flos* extract; the values marked with the same superscripts reveal statistically significant differences (*p* < 0.05).

**Table 2:** The plant extract cytotoxicity on *Artemia salina* larvae.

Sample	Concentration (μg/mL)								
*Control*									
Concentration	50	100	200	400	800	1200	1500–2400	2500–3400	>3500
BSL%	0	0	0	0	0	0	0	0	0
*Calendulae flos* extract									
Concentration	50	100	200	400	800	1200	1590	2500	-
BSL%	0	0	0	0	0	0	0	0	-
*Ginkgo bilobae folium* extract									
Concentration	50	100	200	400	800	1200	1770	2800	-
BSL%	0	0	0	0	0	0	29 ± 5.25 ^a, x^	60 ± 10.70 ^a, y^	-
*Sophorae flos* extract									
Concentration	50	100	200	400	800	1200	1610	3300	5500
BSL%	0	0	0	0	0	0	9 ± 1.38 ^x^	11 ± 2.83 ^y^	14 ± 4.1
*Overview*									
Plant Extract	Minimal lethal concentration tested		Maximal lethal concentration tested			BSL% at 1200 µg/mL		Observations	
	Value (µg/mL)	BSL%	Value (µg/mL)		BSL%				
CE	NA	0	2500		0	0		No toxic	
GE	1770	29	2800		60	0		No toxic	
SE	1610	9	5500		14	0		No toxic	

CE—*Calendulae flos* extract, GE—*Ginkgo bilobae folium* extract, SE—*Sophorae flos* extract, BSL%—Brine shrimp lethality %, NA—not applicable; the values marked with the same superscripts reveal statistically significant differences (*p* < 0.05).

**Table 3:** Predicted cell membrane binding affinities (ΔG) values and permeability coefficients (LogPerm).

Compound	ΔG (kcal/mol)	LogPerm
Bilobalide	−1.92	−7.62
Bilobetin	−3.60	−9.09
Calendoflavoside	−3.14	−17.72
Calenduloside E	−6.11	−6.89
Calenduloside F	−5.83	−8.78
Calenduloside G	−7.02	−12.89
Calenduloside H	−6.72	−12.36
Chlorogenic acid	−2.19	−11.74
Ginkgolide A	−2.38	−7.54
Ginkgolide B	−2.97	−8.42
Ginkgolide C	−2.55	−8.65
Isorhamnetin	−3.40	−7.01
Quercetin	−2.67	−9.84
Rutin	−3.30	−18.13
Sophoricoside	−3.31	−12.26

**Table 4:** Predicted skin permeability coefficients, subcellular localization, and toxicity parameters (activities and probabilities) for selected phytochemicals.

Property	BBD	BBT	CDE	CDF	GKA	GKB	GKC	IRT	QCT
Skin permeability (log Kp)	−2.759	−2.735	−2.735	−2.735	−2.762	−2.737	−2.735	−2.735	−2.735
Subcellular localization	mit.	mit.	mit.	mit.	mit.	mit.	mit.	mit.	mit.
Mitochondrial toxicity	+(0.86)	+(0.63)	+(0.63)	−(0.61)	+(0.75)	+(0.80)	+(0.66)	+(0.65)	+(0.59)
Cytotoxicity	−(0.66)	−(0.93)	−(0.80)	−(0.81)	−(0.62)	−(0.64)	−(0.65)	−(0.95)	−(0.99)
Mutagenicity	+(0.50)	−(0.81)	−(0.93)	−(0.94)	−(0.53)	−(0.56)	−(0.63)	−(0.94)	+(0.51)
Carcinogenicity	−(0.64)	−(0.65)	−(0.58)	−(0.72)	−(0.65)	−(0.60)	−(0.55)	−(0.68)	+(0.68)
Immunotoxicity	+(0.55)	+(0.84)	+(0.99)	+(0.99)	+(0.96)	+(0.94)	+(0.77)	+(0.58)	−(0.87)
Hepatotoxicity	−(0.87)	−(0.8)	−(0.88)	−(0.94)	−(0.83)	−(0.80)	−(0.82)	−(0.72)	−(0.69)
Nephrotoxicity	+(0.80)	+(0.49)	−(0.74)	−(0.91)	+(0.76)	+(0.75)	+(0.88)	−(0.79)	−(0.82)
Respiratory toxicity	+(0.60)	−(0.56)	−(0.50)	−(0.53)	+(0.62)	+(0.53)	+(0.61)	−(0.57)	+(0.62)
Reproductive toxicity	+(0.67)	+(0.72)	+(0.93)	+(0.89)	+(0.67)	+(0.64)	−(0.54)	+(0.84)	+(0.77)
Skin sensitization	−(0.83)	−(0.95)	−(0.80)	−(0.91)	−(0.83)	−(0.81)	−(0.81)	−(0.92)	−(0.74)
Honeybee toxicity	−(0.89)	−(0.72)	−(0.79)	−(0.73)	−(0.81)	−(0.81)	−(0.65)	−(0.88)	−(0.87)
Crustacea toxicity	+(0.56)	−(0.58)	−(0.56)	−(0.65)	−(0.50)	−(0.52)	+(0.51)	−(0.50)	−(0.53)
Fish toxicity	+(0.83)	+(0.88)	+(0.99)	+(0.98)	+(0.92)	−(0.39)	−(0.39)	+(0.83)	+(0.91)
Rat LD_50_ (mg/kg)	90	4000	1750	n.d.	500	500	500	5000	159

BBD—bilobalide, BBT—bilobetin, CDE—calenduloside E, CDF—calenduloside F, GKA—ginkgolide A, GKB—ginkgolide B, GKC—ginkgolide C, IRT—isorhamnetin, QCT—quercetin, LD_50_—median lethal dose.

**Table 5:** Selected predicted activities after screening with the PASS algorithm.

	Apoptosis Inducer		Caspase-3 Stimulant		Caspase-8 Stimulant		c-Myc Inhibitor	
Ligand	Pa	Pi	Pa	Pi	Pa	Pi	Pa	Pi
Bilobalide	0.416	0.066	0.257	0.222	0.386	0.061	0.680	0.003
Bilobetin	0.836	0.006	0.649	0.014	0.464	0.026	0.268	0.128
Calenduloside E	0.876	0.005	0.987	0.002	0.964	0.000	0.689	0.003
Calenduloside F	0.861	0.005	0.992	0.001	0.983	0.000	0.672	0.003
Ginkgolide A	-	-	-	-	-	-	0.608	0.004
Ginkgolide B	-	-	-	-	-	-	0.608	0.004
Ginkgolide C	-	-	-	-	-	-	0.515	0.012
Isorhamnetin	0.880	0.005	0.656	0.013	0.474	0.023	0.316	0.082
Quercetin	0.887	0.005	0.499	0.028	0.428	0.039	0.290	0.104

Pa—the probability of being active; Pi—the probability of being inactive.

**Table 6:** Predicted binding energies and ligand efficiencies (LE) following molecular docking simulations.

	Caspase-3		Caspase-8		c-Myc/Max	
Ligand	ΔG (kcal/mol)	LE	ΔG (kcal/mol)	LE	ΔG (kcal/mol)	LE
Bilobalide	−7.399	0.3217	−7.850	0.3413	−6.225	0.2707
Bilobetin	−9.921	0.2420	−10.145	0.2474	−7.134	0.1740
Calenduloside E	−11.182	0.2485	−9.618	0.2137	−6.258	0.1391
Calenduloside F	−9.336	0.1667	−10.467	0.1869	−6.489	0.1159
Ginkgolide A	−8.517	0.2937	−8.126	0.2802	−6.559	0.2262
Ginkgolide B	−8.364	0.2788	−7.802	0.2601	−6.368	0.2123
Ginkgolide C	−8.128	0.2622	−8.031	0.2591	−6.424	0.2072
Isorhamnetin	−7.848	0.3412	−7.745	0.3367	−5.907	0.2568
Quercetin	−7.828	0.3558	−7.624	0.3465	−5.816	0.2644

## Data Availability

The data are available in the present article and Supplementary Material.

## Supplementary Materials

The following supporting information can be downloaded at: https://www.mdpi.com/article/10.3390/pharmaceutics15082125/s1, Principal Component Analysis.
